# 1188. Evaluation of Antimicrobial Stewardship Discontinuation of Antimicrobial Therapy for Misdiagnosed Urinary Tract Infections in Hospitalized Patients

**DOI:** 10.1093/ofid/ofad500.1028

**Published:** 2023-11-27

**Authors:** Jessica A Duke, Brittany N White, Cyle White

**Affiliations:** Erlanger Health System, Chattanooga, Tennessee; Erlanger Health System, Chattanooga, Tennessee; Erlanger Health System, Chattanooga, Tennessee

## Abstract

**Background:**

Urinary tract infections (UTIs) are often misdiagnosed resulting in unnecessary treatment of asymptomatic bacteriuria (ASB) and pyuria. This practice has been associated with the development of antimicrobial resistance and increased risk of antibiotic-related adverse events, including *Clostridioides difficile* infection (CDI).

**Methods:**

This single-center, retrospective, observational review assessed the effect of a unique antimicrobial stewardship initiative to reduce inappropriate antibiotic use in patients with misdiagnosed UTIs. In July 2018, Erlanger Health System revised the antimicrobial stewardship policy to include automatic discontinuation of antibiotics for patients with misdiagnosed UTIs. Adult patients admitted from November 2017 to November 2022 who received an antibiotic order for ASB or pyuria were included. Presence of UTI symptoms was evaluated via comprehensive chart review. The intervention group consisted of patients with ASB in which antibiotics were discontinued by the antimicrobial stewardship team. Patients with altered mental status (AMS) were included. The primary endpoint was days of antimicrobial therapy. Secondary outcomes included 30-day hospital readmission, hospital mortality, incidence of CDI, hospital length of stay (LOS), and growth of multidrug-resistant organisms in subsequent urine cultures.

**Results:**

A total of 306 patients were included in this study. After propensity score matching, 70 patients were included in each group. The median age was 74 ± 10 years, 85.7% were female, and 55% presented with AMS. Patients in the intervention group received a median of 2 days of antibiotics compared to 6 days in the pre-intervention control (p< 0.0001). Hospital LOS was longer in the intervention group (6 vs 4 days, p< 0.0001). There were no significant differences in other secondary outcomes. Antibiotics were reinitiated in 7% of patients.

**Conclusion:**

This antimicrobial stewardship initiative was associated with a reduction in inappropriate antibiotics days for misdiagnosed UTIs. This intervention is novel as most interventions described are passive and rely on recommendations to providers. The findings of this evaluation highlight the effectiveness of active interventions on reducing inappropriate antimicrobial use.

**Disclosures:**

**All Authors**: No reported disclosures

